# Feasibility of Telaprevir-Based Triple Therapy in Liver Transplant Patients with Hepatitis C Virus: SVR 24 Results 

**DOI:** 10.1371/journal.pone.0080528

**Published:** 2013-11-12

**Authors:** Christoph R. Werner, Daniel P. Egetemeyr, Ulrich M. Lauer, Silvio Nadalin, Alfred Königsrainer, Nisar P. Malek, Christoph P. Berg

**Affiliations:** 1 Medical Clinic, Department of Gastroenterology, Hepatology, and Infectiology, University Hospital Tuebingen, Tuebingen, Germany; 2 Department of General, Visceral and Transplant Surgery, University Hospital Tuebingen, Tuebingen, Germany; Kaohsiung Medical University Hospital, Kaohsiung Medical University, Taiwan

## Abstract

Management of recurrent Hepatitis C virus (HCV) infection following liver transplantation remains a major challenge. In non-transplanted HCV genotype 1 patients, the introduction of protease inhibitor-based regimens has significantly increased the rate of sustained virological response. In this follow-up study, on the first published cohort of post-liver transplant patients treated with telaprevir-based triple therapy, we investigated both efficacy and safety data in follow-up to 24 weeks (SVR 24) after end of treatment (EOT). SVR 24 efficacy and safety data from 9 liver transplant HCV patients being treated with telaprevir, pegylated interferon, and ribavirin, showed 5 of the transplanted patients accomplished the full duration of the 48 week triple therapy.

Notable were the 4 patients found to be HCV RNA-negative at week 4, and 8 patients at week 12. Upon EOT, at week 48, 6 patients were HCV RNA-negative. Importantly, at follow-up (24 weeks after EOT), a favorable sustained virological response rate was observed in 5 of these patients with HCV RNA remaining negative, including in one patient who discontinued treatment prematurely. Due to side effects, 2 patients discontinued, 2 suffered from virological breakthrough after the telaprevir treatment phase, and 1 patient had a relapse after EOT. Two thirds of patients exhibited hematological side effects requiring ribavirin dose reductions, administration of erythropoetin, or even blood transfusions.

This retrospective analysis provides evidence that - with respect to SVR 24 - liver transplant patients suffering from HCV genotype 1 recurrence may benefit from a telaprevir-based triple therapy as this new regimen showed acceptable antiviral efficacy in this small cohort of mostly pre-treated patients. Management of drug-drug interactions is challenging, but feasible. In part severe side effects are frequent during treatment and require therapeutic interventions.

## Introduction

In 2011 the first direct acting antivirals (DAA), the drug class of protease inhibitors (PI) represented by boceprevir and telaprevir (TVR), were approved by the authorities for treatment of chronic Hepatitis C virus (HCV) genotype 1 infections. Due to superior antiviral efficacy in combination with pegylated interferon (PEG-IFN) and ribavirin (RBV) their use was quickly adopted in the clinic [1,2] even though management of side effects was more time intensive and costly, compared to the former dual therapy with PEG-IFN and RBV alone. 

Despite this major innovation, difficult-to-treat populations remained; (i) patients with liver cirrhosis, (ii) non-response patients, (iii) elderly patients, and especially (iv) patients with recurrent HCV infection after liver transplantation (LT). 

Following successful treatment of HCV, post-LT patients have been shown to exhibit significantly reduced morbidity rates corresponding to increased survival times [3,4]. The translation of this superior antiviral efficacy of PI-based triple therapies from non-LT to post-LT patients therefore constitutes a very attractive goal [5-7]. However, there were concerns regarding any post-LT application of PI-based triple therapies. The main concerns were the potentially complex drug-drug interactions between immunosuppressive agents and PI due to the shared metabolic pathway CYP 3A4 [8], possibly resulting in over or under-dosing of both competing drugs and resulting in a loss of efficacy or increased toxicity/ side effects of these agents. 

However, in recent work we have been able to demonstrate that management of a TVR-based triple therapy regimen in patients post-LT is feasible with respect to drug-drug interactions, although side effects were common [9]. In this small pilot study the viral response was shown to be excellent with no treated patients showing any detectable HCV viral load at the end of the TVR phase of the TVR-based triple therapy (treatment week 12)[9].

The aim of this follow-up study is to retrospectively evaluate the antiviral efficacy and safety of the TVR triple therapy in post-LT patients with respect to sustained viral response rates 24 weeks (SVR 24) after planned discontinuation of PEG-IFN and RBV (week 48 of treatment). 

## Patients and Methods

In this study, 9 patients who were being consecutively treated for the recurrence of HCV genotype 1 post-LT were included [9]. The mean age of the patients was 60.9 years and the majority of patients were male (7 patients). All patients had a liver biopsy at least once post-LT and the median Ishak fibrosis score was 2 (the maximum possible score was 6). Liver function post-LT was compensated, except for 1 patient who suffered from a cholestatic recurrence of HCV. The immunosuppressive regimens were heterogeneous: 4 patients received tacrolimus (TAC), 4 patients were on cyclosporine A (CSA), and 1 patient received sirolimus (SIR) as the main immunosuppressive agent. 4 patients were given mycophenolate mofetil (MMF) as co-medication. 5 patients received low-dose steroids (2.5 - 5 mg/day; Table 1). 7 patients with HCV genotype 1b and 2 patients with HCV genotype 1a were included in our study. As for the IL28B gene polymorphism, 8 of the 9 patients had the CT genotype; 1 patient exhibited the most unfavorable TT genotype. 7 study patients had received antiviral treatment at least once before LT, and 8 patients had undergone antiviral treatment at least once post-LT; notably, none of these patients had been able to achieve a sustained clearance of HCV (Table 1). Two patients were on low-dose PEG-IFN at the beginning of our post-LT triple therapy (patients 3 and 5; Table 1). Another patient had suffered from a cholestatic recurrence of an HCV infection just shortly post-LT (initial viral load 23.9 Mio IU/ml) and therefore was set on a PEG-IFN plus RBV dual therapy. Then, due to a primary viral non-response with a rising viral load after having reached a nadir, TVR was added after 1 month of this dual therapy (patient 8; Table 1). 

**Table 1:** Individual characteristics of the 9 patients included in this study (updated and modified after [9]).

**Patient**	**#1**	**#2**	**#3**	**#4**	**#5**	**#6**	**#7**	**#8**	**#9**
**Selected individual characteristics**									
Gender	male	female	male	female	male	male	male	male	male
Age at baseline (years)	51	59	53	70	58	67	61	58	71
Immunosuppression	Tac, Ster	CsA, Ster, MMF	CsA, Ster, MMF	Sir, Ster	Tac	Tac, MMF	CsA, MMF	CsA, Ster	Tac
Post-LT Ishak fibrosis score*	2	3	2	1	5	2	2	0	3
HCV genotype	1a	1a	1b	1b	1b	1b	1b	1b	1b
IL28B genotype	CT	TT	CT	CT	CT	CT	CT	CT	CT
**HCV treatment history**									
Pre-LT treatment; response	PEG-IFN, RBV; NR	PEG-IFN, RBV; NR	-	PEG-IFN, RBV; REL	IFN, RBV; NR (then: low dose PEG-IFN)	IFN, RBV, amanta-dine; NR (then: low dose PEG-IFN)	PEG-IFN, RBV (disc. due to subject-tive side effects)	PEG-IFN, RBV; NR	-
Post-LT treatment; response	PEG-IFN, RBV; NR	PEG-IFN, RBV; NR	PEG-IFN, RBV; NR twice (then: low dose PEG-IFN)	PEG-IFN, RBV; REL	PEG-IFN, RBV; NR (then: low dose PEG-IFN)	-	PEG-IFN, RBV; BT	PEG-IFN, RBV; NR (then: “add-on” TVR)	PEG-IFN; NR
**Details of antiviral treatment, dose reductions**										
PEG-IFN dose (µg)^§^		180 (2a)	180 (2a)	180 (2a)	180 (2a)	80 (2b)	180 (2a)	180 (2a)	180 (2a)	180 (2a)
RBV dose at baseline°		1000^‡^	600	1000	1000	800	600	600	1000	1000^†^
RBV dose at TW 12°		-	200	800	1000	800	200 TW 8 - 10; no RBV from TW 10 - 12	400	800	-
RBV dose at EOT°		-	200	1000	1000	800	600	400	800	-
EPO use		no	yes	yes	no	yes	yes	yes	yes	no
Blood transfusions		no	yes	yes	no	yes	yes	yes	yes	no
GC-SF use		no	no	no	no	yes	no	no	yes	no
**HCV response to treatment**										
HCV VL at baseline (IU/ml)^$^		9.3 Mio.	17.9 Mio.	783.000	3.8 Mio.	9.390	341.000	283.000	339.000	3.78 Mio.
HCV VL TW 4 (IU/ml)		< LLOQ, > LLOD^¶^	367	< LLOQ, > LLOD	16	negative	negative	< LLOQ, > LLOD	negative	negative^†^
HCV VL TW 12 (IU/ml)		Positive	negative	negative	negative	negative	negative	negative	negative	-
HCV VL TW 24 (IU/ml)		-	positive (BT TW 28)	Positive (BT TW 16)	negative	negative	negative	negative	negative	-
HCV VL TW 48 (IU/ml)		-	-	-	negative	negative	negative	negative	negative	-
HCV VL SVR 4 (IU/ml)		-	-	-	negative	negative	negative	negative	negative	negative
HCV VL SVR 12 (IU/ml)		-	-	-	positive	negative	negative	negative	negative	negative
HCV VL SVR 24 (IU/ml)		-	-	-	positive	negative	negative	negative	negative	negative
**Therapy status**		Discon-tinued	BT	BT	relapse	SVR 24	SVR 24	SVR 24	SVR 24	SVR 48

* Maximum possible Ishak fibrosis score is 6; ^†^ Discontinued at TW 4 because of subjective side effects of the therapy; ^‡^ Discontinued at TW 2 because of pneumonia; ^¶^ At discontinuation; ^§^ In parentheses: subtype of PEG-IFN; ^$^ In patients with low-dose PEG-IFN or PEG-IFN/RBV treatment: time-point of add-on of TVR; ° In mg/day. Abbreviations: BT: breakthrough; CsA: cyclosporine, HCV: Hepatitis C virus, IFN: interferon, LLOD: lower level of detection, LLOQ: lower level of quantification, Mio: millions; MMF: mycophenolate mofetil, NR: non-response, PEG-IFN: pegylated interferon, RBV: ribavirin, REL: relapse, Ster: steroid, Sir: sirolimus, Tac: tacrolimus, TVR: telaprevir; TW: treatment week; VL: viral load.

This study has been conducted according to the principles expressed in the Declaration of Helsinki. The ethical committee of the Medical Faculty of the University of Tübingen approved this retrospective analysis and waived the need for written informed consent because of the anonymous evaluation of patient data from patient records. Treatment indication was discussed in our interdisciplinary transplant board. As limited experience existed in the post-LT setting, patients were thoroughly informed of possible known interactions and side effects prior to the on-ward treatment in our hospitals outpatient department. 

Patients gave verbal consent, as the used medications (TVR, PEG-IFN, and RBV) were all approved by the authorities for treatment of HCV. Consent was documented in the medical records.

For routine assessment of IL28B genotype, written informed consent was obtained according to German law. 

Adjustment of the individualised doses of immunosuppressants, under TVR-based triple therapy, were undertaken in our hospital during a 6-8 day stay with on-ward surveillance; subsequent outpatient surveillance was organized in collaboration with general practitioners and our outpatient department. In close consultation with our study center, dosages were adjusted to hit the individual target ranges of the different immunosuppressants (CSA, SIR, and TAC). Visits to the outpatient department were scheduled at weeks 4, 8, 12, with additional individual appointments as required. After ending the TVR-based triple treatment, dual therapy with PEG-IFN and RBV was extended until treatment week (TW) 48. After planned discontinuation of TVR, immunosuppressant dosages were set back to the same doses used pre-exposure to TVR and were adjusted again individually in response to measured trough levels. After the first 12 weeks, visits in the outpatient department were scheduled at least on TW 16 to 18, 24, 36, 48, and on demand. After finishing the triple therapy, follow-up visits were scheduled monthly until post-treatment week 12, and then on post-treatment week 24. Thereafter, the patients were seen in individual intervals according to our post-LT routine. During therapy and follow-up, clinical chemistry parameters as well as HCV viral loads (Roche Cobas AmpliPrep/Roche Cobas TaqMan, Roche Diagnostics GmbH, Mannheim, Germany; lower level of quantification (LLOQ) = 15 IU/mL) were measured at TW 0, 4, 8, 12, 16-18, 24, 36, 48, as well as in the follow-up period (at 1, 2, 3, 6 months post treatment), and individually between these dates. Clinical examinations were performed on the same time points and, if needed, in between.

## Results

### Efficacy

The mean time of treatment was 33 weeks (range 2 - 48 weeks). 7 of the 9 patients completed the 12-week phase of TVR-based triple therapy, and 5 of the 9 patients completed the full-treatment course of 48 weeks. In the intention-to-treat (ITT) analysis we found that 4 out of the 9 patients were HCV RNA-negative at TW 4. After 12 weeks of treatment 8 out of 9 patients were HCV RNA-negative, and after 48 weeks of treatment 6 out of 9 patients were negative, including one patient who had discontinued treatment prematurely at TW 4 due to side effects. 

**Figure 1:**
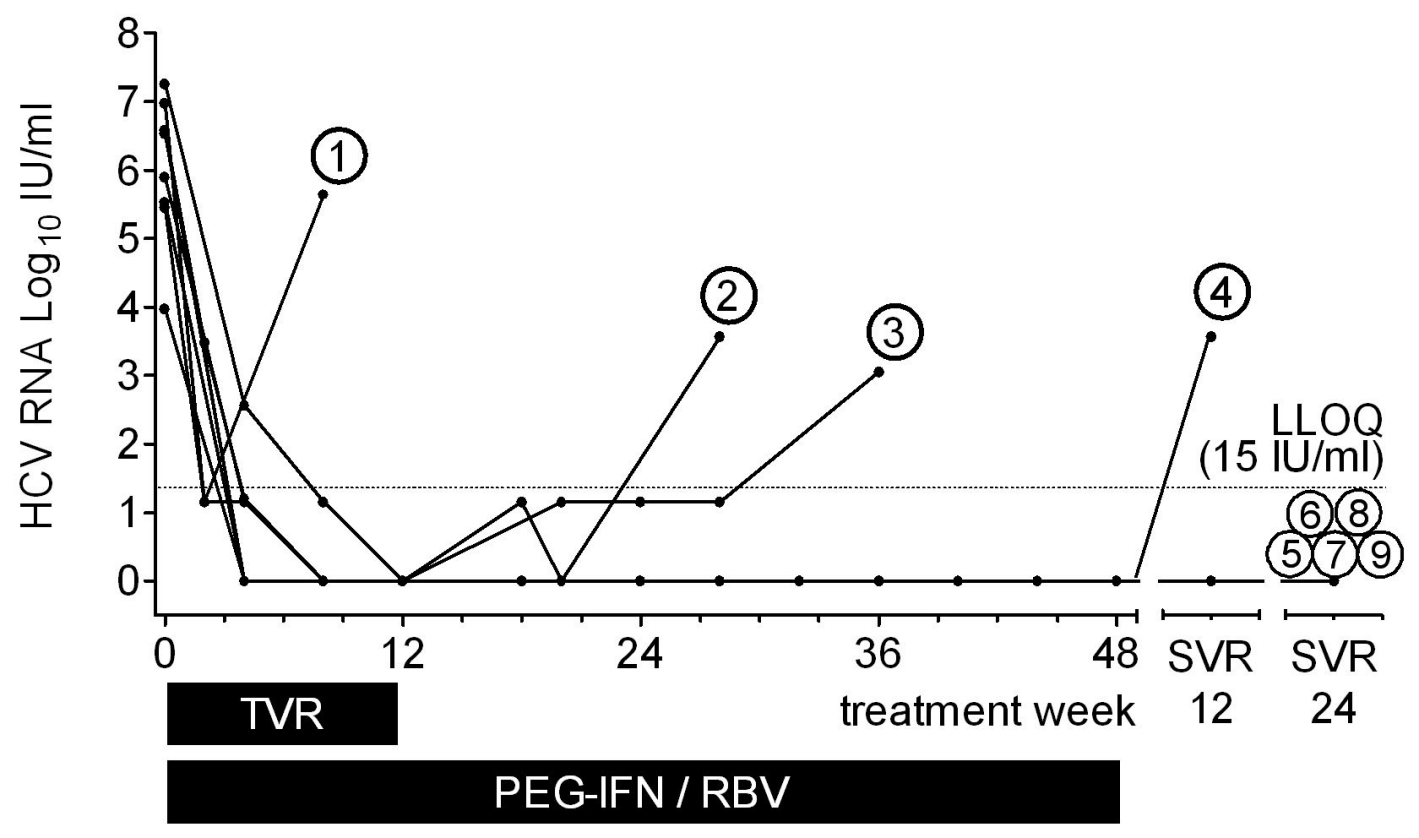
Individual courses of HCV viral load over time. Numbers indicate patient numbers as outlined in Table 1.

At 24 weeks of follow-up (SVR 24) 5 out of 9 patients remained HCV RNA-negative with a mean follow-up time of 30 weeks (range 24 - 56 weeks; see Table 1 and Figure 1 for details). 

In two instances TVR-based triple therapy had to be discontinued: One patient suffered from bacterial pneumonia at week 2 (patient 1; Table 1), and the other patient discontinued at week 4 due to side effects (patient 9; Table 1). The latter was HCV RNA-negative at the time of discontinuation and remained HCV RNA-negative thereafter (follow-up of 56 weeks). Two patients exhibited a virological breakthrough at TW 16 and 28, respectively (patients 3 and 2; Table 1). One patient had a virological relapse between follow-up weeks 4 and 12 (patient 4; Table 1). 

Remarkably, in our cohort 2 out of 5 patients with previous post-LT non-response to PEG-IFN/RBV therapy were now able to achieve a SVR 24 under our TVR-based triple therapy regimen. Of those patients who had not shown a non-response to a post-LT dual therapy (PEG-IFN monotherapy, PEG-IFN/RBV relapse, or breakthrough), or had not received any antiviral therapy post-LT, 3 out of 4 patients achieved a SVR 24. 

Due to the small number of patients in this pilot cohort, it is not possible to determine any positive predictors for SVR. 

### Immunosuppression levels after TVR-based triple therapy

Changes in immunosuppression due to drug-drug interactions during the first twelve weeks of TVR-triple therapy are reported in detail in our recent article [9]. In our cohort, reestablishment of pre-TVR immunosuppressant dosages and readjustment of daily doses according to measured trough levels could be permuted without complications. Importantly, no case of transplant rejection was observed following discontinuation of TVR, which had constituted a major concern before initiation of this study.

### Side effects

In our cohort, no patients were lost during therapy or follow-up, although adverse events occurred in most of the patients. Treatment was ceased due to severe side effects in only one case (at TW 2). Five patients were hospitalized during treatment and follow-up because of adverse events. One of those patients was hospitalized four times for different reasons (patient 8, Table 1). The reasons for in-hospital treatment up until TW 12 were: bacterial pneumonia, TAC overdosing with renal failure, and infectious enteritis with Yersinia pseudotuberculosis. During the second phase of treatment covering the period between discontinuation of TVR (at TW 12) until the end of treatment (EOT) at TW 48, hospitalizations were due to exacerbated diabetes mellitus, raised liver enzyme levels caused by histologically proven non-alcoholic steatohepatitis, and an attack of gout. 

In week 5 of the follow up phase one patient (patient 8, Table 1) was admitted to hospital due to increased liver enzymes (ALT 4 times above upper limit of normal); a liver biopsy revealed a histologically confirmed acute rejection. After treatment with high dose prednisolone and addition of MMF, liver enzyme levels decreased. Thereafter, the patient suffered from a reactivation of Cytomegalovirus (CMV) with viral DNA being positive in both plasma and leukocytes. Following pre-emptive treatment with valganciclovir this patient’s liver enzyme levels normalized. Shortly thereafter, the patient presented with diarrhea at our emergency room, with a Norovirus and Clostridium difficile co-infection, along with a Proteus spp. urinary tract infection. In the course of a calculated antibiotic and symptomatic treatment, the patient recovered completely.

6 of the 9 patients had anemia with a hemoglobin level lower than 8.5 g/dL. 6 of the 9 patients were treated with erythropoetin (EPO), and the same number of patients needed blood transfusions. Only one patient, who was treated for more than 4 weeks, received neither growth factors nor blood transfusions. Two other patients, who did not receive EPO or blood transfusions, discontinued at TW 2 and TW 4, respectively. 5 of the 9 patients required RBV dose reductions because of anemia during the first twelve weeks, although in two patients the RBV dose could be raised again after discontinuation of TVR (see Table 1 for details). 8 of the 9 patients had leukocyte levels lower than 2,500/µL and 4 of the 9 patients had leukocyte counts less than 1,500/µL during therapy. Two of the 9 patients needed granulocyte colony-stimulating factor (GC-SF) at least once. Five of the 9 patients had platelet counts less than 50,000/µL with no apparent clinical side effects. Six patients developed increases in serum creatinine levels exceeding 1.5 mg/dL.

## Discussion

In this retrospective single center study we have analyzed the antiviral efficacy and safety of a TVR-based triple therapy in a small cohort of post-LT patients with HCV genotype 1 recurrence. In our cohort, of mostly pre-treated patients, we were able to demonstrate a substantial rate of sustained viral response at 24 weeks (SVR 24) after EOT (5 out of 9 patients). 

Obviously, our cohort is small in number therefore comparison with larger, prospective trials is of limited significance. However, our results suggest that it is possible to achieve higher SVR rates than those previously obtained with a dual therapy consisting of PEG-IFN/RBV in a post-LT setting: in a meta-analysis including all genotypes pooled SVR rates have to date only reached 30% (range 8 - 50%) [10,11]. Interestingly, our data are in line with an interim analysis provided by the multicentric CRUSH-C post-LT cohort presented by Verna et al. at the recent meeting of the European Association for the Study of the Liver (EASL) 2013 [12]: here, SVR 4 rates of 41% (7 out of 17 patients) were achieved, which exceed data provided by the French post-LT experience [13], showing SVR 12 rates of 20% (1 out of 5 patients) in the TVR subgroup. In the CRUSH-C study all patients who achieved SVR 4 had been HCV RNA-negative at TW 4 [12]. 

In our study, most of the patients with SVR 24 were HCV RNA-negative at TW 4, thus emphasizing the exceptional significance of a rapid viral response for treatment success also in the post-LT setting. Importantly, in our cohort, 2 out of 5 patients with previous non-response to PEG-IFN/ RBV post-LT also achieved a SVR 24. 

The major side effect of TVR-triple therapy is hematological toxicity with two thirds of patients suffering from severe anemia, thus requiring blood transfusions and administration of EPO. Both Verna et al. (49% transfusion, 87% growth factors; [12]), and Coilly et al. (85% anemia, 96% growth factors; [13]) found similar rates of anemia and a need for the application of growth factors in their cohorts. Accordingly, in an interim analysis of patients undergoing TVR or boceprevir triple therapy post-LT, 77% of patients in the TVR group received EPO [14]. In the French post-LT cohort, discontinuation due to side effects occurred in 23% [13], which corresponded to the range observed in our cohort (22%). During treatment, especially in the early post-TVR period, no acute rejections occurred. This finding could be confirmed in the CRUSH-C group, with a low frequency of acute rejections during therapy (2 out of 101; [12]). Of note, no patients died in our small cohort, while in the larger multicentric cohorts treatment-associated mortality rates were reported to be in the range of 2-4% [12-14]. 

In our retrospective analysis, similar to the preliminary results of the CRUSH-C study and the French post-LT experience, the antiviral potency of DAA has been positively shown. To some extent achieving SVR was possible in former non response patients, who otherwise lacked alternate treatment options. However, this surplus in antiviral efficacy is associated with increased rates of side effects which may lead, albeit rarely, to a deleterious outcome [12-14] thus jeopardizing treatment success. 

The first-generation PI TVR and boceprevir are just the vanguard of other forthcoming DAA, including second-generation PI like simeprevir, polymerase inhibitors like sofosbuvir, and NS5A inhibitors like daclatasvir, which will be approved by the authorities in the near future. Moreover, IFN-free treatment regimens will soon be possible by combining these new drug classes. Next-generation DAA show a superior antiviral efficacy and exhibit a far less harmful spectrum of side effects in non-LT patients [15]. Furthermore, sofosbuvir and daclatasvir have been successfully used in a LT patient and not shown significant interference with immunosuppression [16]. Most likely, the treatment of post-LT patients in the future will be dominated by the next-generation DAA, presumably without PEG-IFN, as they seem to be better tolerated and the side effects more readily controlled. 

In conclusion, respecting the limitations described above, TVR-based triple therapy in post-LT patients is feasible and reveals an acceptable efficacy with respect to SVR 24. In part, severe side effects are frequent during treatment and require therapeutic interventions. In addition, management of drug-drug interactions is challenging. Future treatment regimens are likely to lead to less harmful antiviral treatments, however the current first-generation DAA treatment should be indicated cautiously, and be conducted by an experienced treatment center.

## References

[1] Ghany MG, Nelson DR, Strader DB, Thomas DL, Seeff LB (2011) An update on treatment of genotype 1 chronic hepatitis C virus infection: 2011 practice guideline by the American Association for the Study of Liver Diseases. Hepatology 54: 1433-1444. doi:10.1002/hep.24641. PubMed: 21898493. 21898493 PMC3229841

[2] Sarrazin C, Berg T, Cornberg M, Dollinger M, Ferenci P et al. (2012) [Expert opinion on boceprevir- and telaprevir-based triple therapies of chronic hepatitis C]. Z Gastroenterol 50: 57-72. doi:10.1055/s-0031-1282015. PubMed: 22222799. 22222799

[3] Berenguer M, Palau A, Aguilera V, Rayón JM, Juan FS et al. (2008) Clinical benefits of antiviral therapy in patients with recurrent hepatitis C following liver transplantation. Am J Transplant 8: 679-687. doi:10.1111/j.1600-6143.2007.02126.x. PubMed: 18294165. 18294165

[4] Picciotto FP, Tritto G, Lanza AG, Addario L, De Luca M et al. (2007) Sustained virological response to antiviral therapy reduces mortality in HCV reinfection after liver transplantation. J Hepatol 46: 459-465. doi:10.1016/j.jhep.2006.10.017. PubMed: 17196700. 17196700

[5] Jacobson IM, McHutchison JG, Dusheiko G, Di Bisceglie AM, Reddy KR et al. (2011) Telaprevir for previously untreated chronic hepatitis C virus infection. N Engl J Med 364: 2405-2416. doi:10.1056/NEJMoa1012912. PubMed: 21696307. 21696307

[6] Zeuzem S, Andreone P, Pol S, Lawitz E, Diago M et al. (2011) Telaprevir for retreatment of HCV infection. N Engl J Med 364: 2417-2428. doi:10.1056/NEJMoa1013086. PubMed: 21696308. 21696308

[7] Sherman KE, Flamm SL, Afdhal NH, Nelson DR, Sulkowski MS et al. (2011) Response-guided telaprevir combination treatment for hepatitis C virus infection. N Engl J Med 365: 1014-1024. doi:10.1056/NEJMoa1014463. PubMed: 21916639. 21916639 PMC3809077

[8] Garg V, van Heeswijk R, Lee JE, Alves K, Nadkarni P et al. (2011) Effect of telaprevir on the pharmacokinetics of cyclosporine and tacrolimus. Hepatology 54: 20-27. doi:10.1016/S0168-8278(11)60045-7. PubMed: 21618566. 21618566

[9] Werner CR, Egetemeyr DP, Lauer UM, Nadalin S, Königsrainer A et al. (2012) Telaprevir-based triple therapy in liver transplant patients with hepatitis C virus: a 12-week pilot study providing safety and efficacy data. Liver Transpl 18: 1464-1470. doi:10.1002/lt.23542. PubMed: 22941516. 22941516

[10] Berenguer M (2008) Systematic review of the treatment of established recurrent hepatitis C with pegylated interferon in combination with ribavirin. J Hepatol 49: 274-287. doi:10.1016/j.jhep.2008.05.002. PubMed: 18571272. 18571272

[11] Crespo G, Mariño Z, Navasa M, Forns X (2012) Viral hepatitis in liver transplantation. Gastroenterology 142: 1373-1383. doi:10.1053/j.gastro.2012.02.011. PubMed: 22537446. 22537446

[12] Verna EC, Burton JR, O'Leary JG, Lai JC, Saxena V et al. (2013) A Multicenter Study of Protease Inhibitor-Triple Therapy in HCV-Infected Liver Transplant Recipients: Report from the CRUSH-C Group (Abstract #23) J Hepatol 58: S10. doi:10.1016/S0168-8278(13)60025-2.

[13] Coilly A, Dumortier J, Botta-Fridlund D, Latournierie M, Leroy V et al. (2013) End of Treatment Response after Protease-Inhibitor (PI)-based Therapy for Hepatitis C Recurrence after Liver Transplantation: A Multicentric European Experience (Abstract #1422) J Hepatol 58: S572. doi:10.1016/S0168-8278(13)61420-8.

[14] Pungpapong S, Aqel BA, Koning L, Murphy JL, Henry TM et al. (2013) Multicenter experience using telaprevir or boceprevir with peginterferon and ribavirin to treat hepatitis C genotype 1 after liver transplantation. Liver Transpl 19: 690-700. doi:10.1002/lt.23669. PubMed: 23696372. 23696372

[15] Sulkowski M, Gardiner DF, Rodriguez-Torres M, Reddy KR, Hassanein T et al. (2013) Sustained Virologic Response With Daclatasvir Plus Sofosbuvir ± Ribavirin (RBV) in Chronic HCV Genotype (GT) 1-Infected Patients Who Previously Failed Telaprevir (TVR) or Boceprevir (BOC) (Abstract # 1417) J Hepatol 58: S 570.

[16] Fontana RJ, Hughes EA, Bifano M, Appelman H, Dimitrova D et al. (2013) Sofosbuvir and daclatasvir combination therapy in a liver transplant recipient with severe recurrent cholestatic hepatitis C. Am J Transplant 13: 1601-1605. doi:10.1111/ajt.12209. PubMed: 23593993. 23593993

